# Chemometric Assessment of Bulgarian Wastewater Treatment Plants’ Effluents

**DOI:** 10.3390/molecules25194408

**Published:** 2020-09-25

**Authors:** Galina Yotova, Tony Venelinov, Stefan Tsakovski

**Affiliations:** 1 Faculty of Chemistry and Pharmacy, Sofia University “St. Kliment Ohridski”, 1164 Sofia, Bulgaria; G.Yotova@chem.uni-sofia.bg; 2 Faculty of Hydraulic Engineering, University of Architecture, Civil Engineering and Geodesy, 1046 Sofia, Bulgaria; TVenelinov_fhe@uacg.bg

**Keywords:** wastewater treatment plant, water quality, nutrients, chemometrics, load effluent profiles, source apportioning

## Abstract

Surface water quality strongly depends on anthropogenic activity. Among the main anthropogenic sources of this activity are the wastewater treatment plant (WWTP) effluents. The discharged loads of nutrients and suspended solids could provoke serious problems for receiving water bodies and significantly alter the surface water quality. This study presents inventory analysis and chemometric assessment of WWTP effluents based on the mandatory monitoring data. The comparison between the Bulgarian WWTPs and previously reported data from other countries reveals that discharged loads from investigated WWTPs are lower. This is particularly valid for total suspended solids (TSS). The low TSS loads are the reason for the deviations of the typical calculated WWTP effluent ratios of Bulgarian WWTPs compared to the WWTPs worldwide. The performed multivariate analysis reveals the hidden factors that determine the content of WWTP effluents. The source apportioning based on multivariate curve resolution analysis provides detailed information for source contribution profiles of the investigated WWTP effluent loads and elucidate the difference between WWTPs included in this study.

## 1. Introduction

Ten years ago, half of the world’s population lived in urban areas [1] and this is expected to increase to 66% by 2050 mainly due to urban growth and the demographic changes in less developed countries [2]. Estimates are that the global population growth is to reach 10 billion by 2050 due to economic development [3]. Population growth requires increasing access to surface water with sufficient quality and leads to an increase in drinking water scarcity worldwide, but it also leads to an increase of wastewater production, since a substantial part of the freshwater will end up as wastewater [4]. The surface water quality depends on surface/subsurface flows (non-point sources) and on the population density of the area, which determines the composition of the wastewater treatment plants’ (WWTPs) effluents (point sources) [5]. The effluent discharges from WWTPs are comparatively easier to regulate and monitor than the nonpoint sources [6]. This is why the European Union (EU), focused on keeping up to best practices in assessing surface waters quality and efficiency of wastewaters treatment processes, has adopted Directive 91/271/EEC [7]. The treated wastewaters consist of a complex mixture of potential environmental pollutants [8,9] such as organic matter [6], nutrients [10], fine sediments [11] and micropollutants—metals, pesticides, pharmaceutically active compounds, personal care products or illicit drugs [12].

The difference in the concentrations of the regulated parameters in the effluents and in the receiving water bodies determines the effect of WWTPs on the environment [13,14,15]. In recent years, efforts have been directed to reduce nutrient loading [16], but nutrient concentrations in treated wastewaters frequently exceed those in receiving waters, with uncertain effects on the ecology of these aquatic ecosystems [17,18,19]. While it is easier to distinguish between the obvious effect of poorly treated and/or highly concentrated WWTP effluents, the more difficult task is to estimate effects of well treated and/or highly diluted effluents—the temporal changes downstream [20,21], or when comparing water samples located upstream and downstream from the point source [22]. Therefore, the nutrient loads impact on water quality impairment needs to be assessed considering the dilution of each WWTP discharge alone and through cumulative nutrient loads attenuated by dilution and instream nutrient uptake [23].

Many water bodies have very low nutrient concentrations ranges and small shifts in the load can adversely change in community structure [24,25,26]. Therefore, the discharged volume becomes important because even low-nutrient-containing effluent can still deliver large loads because of the total volume of treated water released from WWTPs. Consequently, a lot of quality concerns for water systems in the U.S.A. are directly bound to the nutrient loads [6]. In 2010, the Environmental Protection Agency (EPA) developed a Total Maximum Daily Load (TMDL) to establish targets for nutrient reduction and as a result to reach water quality goals. TMDLs identify the pollutant stressors and indicate the maximum amount of a specific pollutant that a water body can receive without exceeding the safe water quality limits [27].

The relative contribution of N and P loads discharged from WWTPs in Europe decreased continuously in the last 40 years because stringent regulations for wastewater treatment had been introduced [28,29,30,31,32,33]. The role of advanced P removal technologies (e.g., coagulation, flocculation, and decantation) in increasing P sorption onto suspended colloidal matter is the probable reason for altering the bioavailable P in the river downstream [34]. Di Zhang et al. reported much lower dissolved organic carbon (DOC) and chemical oxygen demand (COD) concentrations in the treated wastewater than those upstream, therefore the organic pollutants in the downstream decreased after receiving WWTP effluent [35]. The decreasing trend of DOC and COD downstream might be attributed to the dilution effect and biodegradation because of the higher dissolved oxygen level. The total P concentration of WWTP was much lower and the granular phosphorus adsorbing any particulate matter became deposited as sediment. The results showed that turbidity gradual decreased and particulate pollutants, such as total P and COD, also gradually decreased. In a study by Figueroa-Nieves et al. [36], the values of specific UV absorbance at 254 nm were found to be lower in WWTP effluents than those measured upstream of the WWTP, suggesting that WWTP effluents are contributing labile carbon fractions to receiving river, thus changing the chemical composition of DOC downstream. More than 40% of the nutrient loads in receiving streams came from WWTP effluents, with the effects on NO_3_–N and PO_4_–P loads being the greatest. Along the Bulgarian coast, 68% of the total nitrogen and 15% of the total phosphorus loads into the Black Sea are deposited by the municipal WWTPs. For the 2005–2010 period, WWTPs “Varna” discharged an average of 650 t nitrogen and 130 t phosphorus per year [37]. The discharge of WWTP effluents could simultaneously change some of the water quality parameters of receiving water bodies and provoke different environmental concerns. Bram et al. [38] found that by mixing clarified water and sludge rich water from the settlement tank of the WWTP, the suspended particle concentration was increased eight-fold within 8 h prior to discharge. The increase of suspended particle concentration was in line with increases in turbidity, oxygen demand and total nutrient load (nitrogen, phosphorus). Similarly to the surface water quality assessment, the assessment of WWTP effluents is a complex and multivariate task. Chemometric approaches are frequently used for monitoring wastewater treatment processes but only a few studies are devoted to the chemometric assessment of WWTP effluents and water quality of receiving water bodies [39,40,41,42,43].

The aim of the present study is to use the mandatory monitoring WWTP data to perform: (i) inventory analysis of loads introduced in the receiving water bodies from discharged WWTP effluents and (ii) chemometric assessment of WWTP effluents using discharged monthly loads. To the best of our knowledge, the proposed multivariate statistical assessment of WWTP effluents including source apportioning is undertaken for the first time in this study.

## 2. Results

### 2.1. Basic Statistics

The dataset used for this study contains information about the flow rate and the concentrations of five water quality indicators (chemical oxygen demand (COD), biochemical oxygen demand (BOD), total N (N), total P (P), total suspended solids (TSS)) obtained in the WWTPs’ routine monitoring in the effluent wastewater. In this study, we selected representative Bulgarian WWTPs, which include all the biggest plants (>50,000 population equivalents—p.e.) and the small plants that discharge in all the biggest rivers, in small rivers and into the Black Sea. The average month concentrations and loads for 39 WWTPs taken throughout the entire 2017 year were calculated. The basic statistics of the obtained dataset are presented in Table 1. Additionally, the calculated loads per p.e. for each one of the 39 WWTPs studied are shown in Table S1 in Supplementary Material.

### 2.2. Multivariate Analysis

The input dataset contains the average month loads (kg/day) for 2017 of COD, BOD, total N, total P and TSS for the studied WWTPs. Thus, the obtained data matrix consists of five columns (water quality indicators) and 468 rows (12 records for each one of the 39 WWTPs).

**Figure 1:**
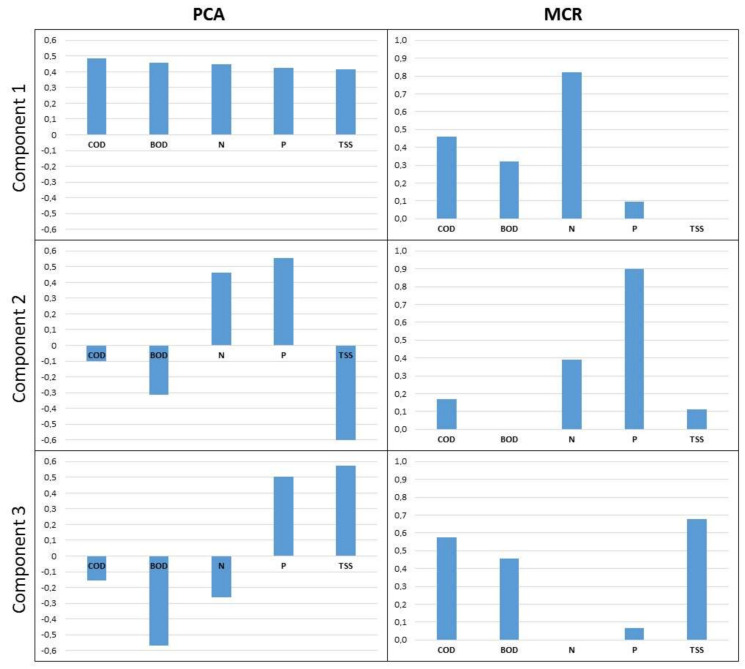
Principal component analysis (PCA) and multivariate curve analysis (MCR) component loadings.

Component loadings obtained by principal component analysis (PCA) and multivariate curve resolution (MCR) are presented in Figure 1. The explained variance of input dataset by the three selected components for PCA and MCR is 97.03% and 98.09%, respectively.

The first PCA component (80.36% of explained variance) describes the average WWTP effluent loads for the investigated WWTPs. The second component (12.31% of explained variance) resembles the positive correlation between TSS, COD and BOD. The last PCA component (4.37% of explained variance) reflects the relation between N load and oxygen-demanding loads.

The first MCR component (36.44% of explained variance) provides the relation between the soluble part of nutrient loads (N, P) and oxygen demand (COD, BOD) and could be conditionally named “soluble nutrients”. The second MCR component (35.56% of explained variance) reflects the relationship between non-biodegradable part of N, P and TSS and could be conditionally named “refractory loads”. The third MCR component (26.09% of explained variance) with the conditional name “suspended solids” presents the relation between TSS and oxygen demanding loads.

**Figure 2:**
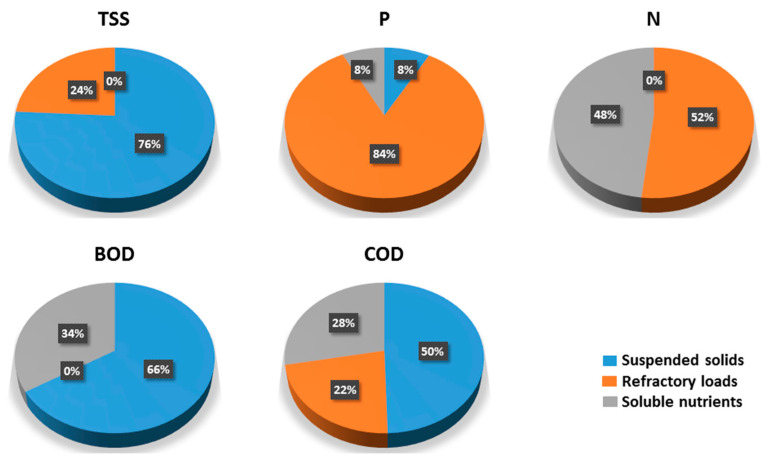
Average source contributions to WWTP effluent loads.

The MCR components provide more chemical meaningful load sources and are used for the source apportioning of WWTP effluent loads. The contribution of each source to each WWTP effluent load based on the whole dataset is presented in Figure 2.

**Figure 3:**
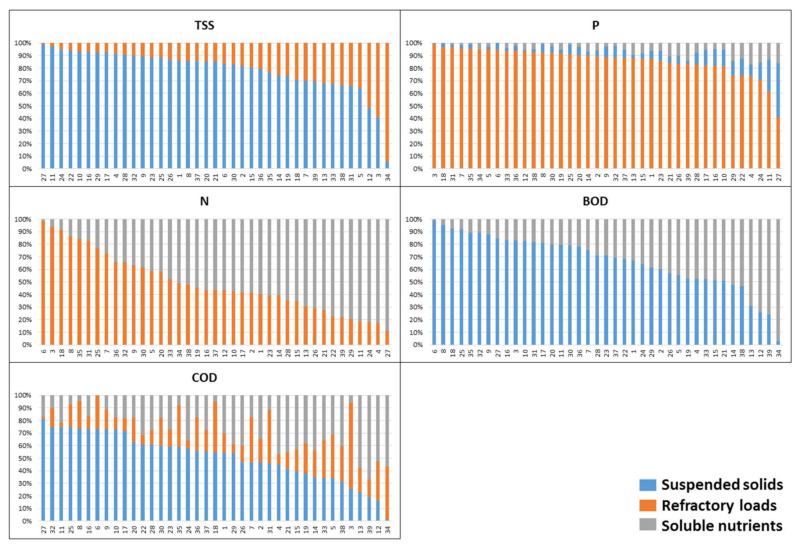
Source contributions to WWTP effluent loads for each WWTP.

To outline the differences between the investigated WWTPs the component scores are used for calculation of the source contribution to WWTP effluent loads of each treatment plant (Figure 3).

## 3. Discussion

### 3.1. Basic Statistics

The mean concentrations in the effluents (n = 468) of the studied WWTPs (39) are lower than the respective limits set in the Directive 91/271/EEC for all the mandatory parameters throughout 2017. This compliance shows accurate treatment operations in all the studied WWTPs. Still, occasionally, some samples exceed the limits (Table S2 in Supplementary Material). These results show the necessity of reconstruction and modernization of the WWTPs of 11-Shumen and 27-Lozenets and their lack of adequate treatment of nitrogen, phosphorus, TSS and organic substances. Inadequate treatment and therefore elevated concentrations of nutrients (N and P) are found in WWTPs without nitrogen removing facilities and chemical precipitation of phosphorus (12-Shabla, 13-Kavarna, 33-Plovdiv, 34-Pazardzhik and 38-Pernik).

The estimated loads per p.e. for the five water quality indicators are in good agreement with the reported by Henze et al., 2008 [4] range variations in person load (COD, BOD, N, P) and with the typical load values (BOD, N, P, TSS) for domestic wastewater reported for the Grand River watershed [44] (see Table 2). Exceedings are observed only for 2 of the 39 WWTPs studied-12-Shabla (N, P) and 13-Kavarna (N). Additionally, a comparison for the five water quality parameters with a study, in which 32 small (400 < p.e. < 4000) WWTPs were included [45], indicates problems only for N and P in 12-Shabla. The personal loads for 12-Shabla are at least two times higher than the rest of the WWTPs (see Table S1 in Supplementary Material) and are excluded from the calculated range, as being an obvious outlier. It could be outlined that the WWTPs, where most problems were found are mainly the plants constructed for lower inlet person load-12-Shabla (360 p.e.), 27-Lozenets (3000 p.e.) and 13-Kavarna (3583 p.e.). It should be noted that the WWTPs in Shabla and Kavarna are currently under reconstruction.

Additionally, a comparison between the person load in Bulgaria (based on this study) and in various countries (based on Henze et al., 2002 [46]) is presented in Table 3. It is easy to notice that the maximum load values for N and P in this study are generally the same as those for the other countries. The minimum values for N and P as well as the load ranges for BOD and especially for TSS are much lower than those for the other countries.

The comparison of the total loads for WWTP-Varna for 2017 with the only published study for Bulgarian WWTPs plants’ loads (WWTP-Varna for the period 2005–2010) [37] shows a decrease of N and P loads 2.4-fold and 5.2-fold, respectively. The reason might be attributed to the general trend in the decrease of the Bulgarian population and the drop in the industrial water discharges to the municipal WWTPs due to abolishment of unprofitable manufactures and the introduction of environmentally friendly technologies. This might be the explanation for all the Bulgarian loads being generally lower than the ones in other countries (Table 3).

### 3.2. WWTP Effluents Ratios

Typical ratios between the COD, BOD, P and TSS for Bulgarian wastewater effluents are calculated and presented in Table 4. The values are obtained by the yearly loads’ ratios for each one of the 39 WWTP.

The incorporation of the P in the biomass is in the heart of the biological phosphorus removal. This results in a reduction of the TSS in the effluent waters. The loss of TSS will increase the typical ratio mgP/mgTSS (0.02–0.07 [47]), which for the studied Bulgarian WWTPs is 0.17. Calculations for nearly 70% of the WWTPs fall outside the ranges for the typical ratio, including plants with high P loads (>0.3 g/p.e./day, Table S1), such as 19-Devnya, 33-Plovdiv, 34-Pazardzhik, etc., as well as plants with low loads (<0.1 g/p.e./day, Table S1), such as 2-Pleven, 14-Balchik, 25-Meden Rudnik and 31-Sopot.

The oxygen demand is primarily generated by the biodegradable solids since the bacteria can only assimilate the organic substrate, for which the oxygen is needed. The inorganic solids and the other inert solids are not used by the bacteria [47]. The part of the TSS which is biodegradable and will represent BOD is referred to as volatile suspended solids (VSS). The reported typical ratio mgBOD/mgTSS is in the range 0.45–0.65 [47]. As seen in Table 4, the average BOD/TSS ratio is 1.05 for the Bulgarian WWTPs. This higher ratio could be attributed to the relatively low TSS loads for all the stations and the additional loss of the TSS due to the biomass incorporation. Little above 50% of the studied stations are calculated to be outside the typical ratio with 25% from them being lower and the remaining 75% above.

The rapidly biodegradable COD is usually 15% to 30% of the total COD [48]. Our results show that 28% (0.28) on average is the ratio between BOD and COD in the final effluents. Half of the studied plants fall outside the typical ratio. Of them, 75% show a higher ratio than 0.3% and 25% show lower mgBOD/mgCOD ratio than 0.15. The higher ratio is characteristic for the Black Sea WWTPs and can be explained with the treatment of primarily domestic waters especially in the summer months (12-Shabla, 14-Balchik, 27-Lozenets, etc.). The lower range is characteristic for WWTPs in industrialized cities–7-Veliko Tarnovo, 24-Burgas and 25-Meden Rudnik.

Based on the ratio calculations, there are 13 out of 39 (33%) that fall within the ranges of the typical ratios for P/TSS, BOD/TSS and BOD/COD.

### 3.3. Multivariate Analysis

The average source contribution to loads based on MCR components, which are responsible for WWTP effluent composition presents valuable information concerning load effluents of the investigated Bulgarian WWTPs (see Figure 2). The estimated average source contribution of TSS is in good agreement with von Sperling and de Lemos Chernicharo [47] where the typical content of biodegradable VSS is 72% of TSS. For the Bulgarian WWTPs, this value is 76% as the remaining 24% is contributed by the “refractory loads” source. The source contribution of P load is dominated by the “refractory loads” source (84%) since the N load is divided by the “refractory loads” and “soluble nutrients” sources. The “suspended solids” source representing the relatively low TSS loads of WWTP effluents contributes to 66% of BOD and 50% of COD load. The other half of the COD load is divided by “soluble nutrients” (28%) and “refractory loads” (22%) sources.

The source contribution to loads of each WWTPs reveals their different source load profiles and provides more detailed information about the discharged WWTP effluents (Figure 3). The presented TSS source profile outlines three WWTPs, which possess a contribution of “refractory loads” higher than 50%. Two of these WWTPs (34-Pazardzhik and 12-Shabla) have only mechanical and biological wastewater treatment. For the third WWTP (3-Lovech), the reason for the high “refractory loads” contribution could be found in the receiving inlet wastewaters from neighbouring industrial zones. The same WWTPs among the others already mentioned with a high P/TSS ratio are characterized by “refractory loads” contribution to P loads higher than 90%. It is expected that the highest “suspended solids” contributions to P load are for WWTPs 27-Lozenets, 11-Shumen and 24-Burgas which possess the lowest “refractory loads” contribution to their TSS loads. The presented source profiles of N loads resemble the big difference between investigated WWTPs. The first group includes the WWTPs 6-Gabrovo, 3-Lovech and 18-Beloslav with “refractory loads” contribution higher than 90% and the other—the WWTPs with dominant “soluble nutrients” contribution (27-Lozenets, 4-Troyan, 24-Burgas and 11-Shumen). Further, this second group of WWTPs is characterized by low contributions of “refractory loads” to TSS and P loads. The different structure of BOD WWTP profiles is similar to those of N loads. The WWTPs with the highest “suspended solids” contribution (6-Gabrovo, 8-Gorna Oryahovitsa and 18-Beloslav) also possess the high impact of “refractory loads” to the N and P loads. The lowest “suspended solids”, respectively the highest “soluble nutrients” contribution to the BOD load is at 34-Pazardzhik, which is characterized by elevated N and P loads. It is obvious that the integral character of COD will lead to the source profiles which reflect the source contributions of the already discussed source contributions. The highest impact of: (i) “suspended solids” possess a seaside resort WWTP at 27-Lozenets; (ii) “refractory loads” possess the WWTP at 3-Lovech and (iii) “soluble nutrients” possess the biggest Bulgarian WWTP at 39-Sofia.

## 4. Materials and Methods

### 4.1. Data Acquisition and Input Data Arrangement

The mandatory monitoring of WWTPs in Bulgaria depends on the class-size of the plant, based on the contributions from the industries to the wastes and population in the community served within the sewer-shed, expressed as the population equivalents (p.e.). Directive 91/271/EEC [7] establishes the requirements for the discharges of five water quality parameters (COD, BOD, TSS, N and P), sampling strategy, and for the minimum annual number of samples collected according to the size of the treatment plant at regular intervals during the year, namely 12 samples for WWTPs with 10,000 to 49,999 p.e. and 24 samples for WWTPs with >50,000 p.e.

**Figure 4:**
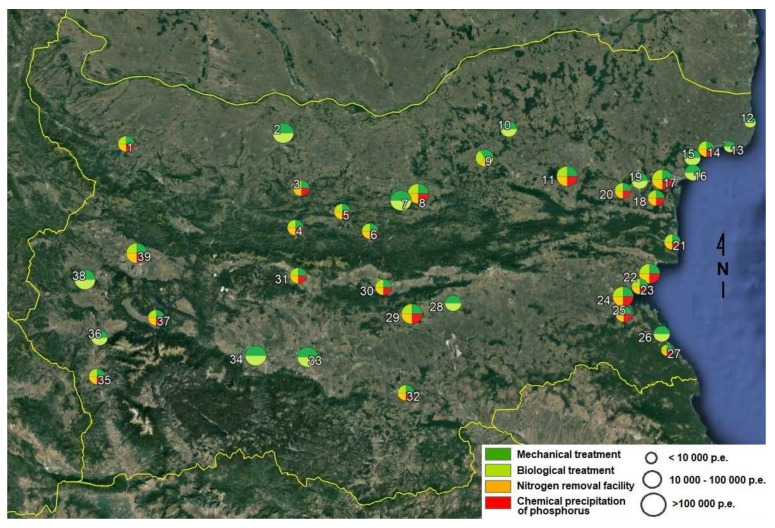
Sampling map.

Mandatory monitoring data were obtained by the Ministry of Regional Development and Regional Works through the Union of Water Supply and Sewerage Operators in the Republic of Bulgaria. Data from 39 WWTPs (Figure 4) were collected for 2017 for the concentrations of COD, BOD, TSS, N and P. The mean monthly concentrations were multiplied by the mean monthly flow rates to calculate the loads. In the case of small WWTPs, the only monthly value for every parameter and the flow rate for the day of the measurement were used for the calculation.

### 4.2. Chemical Analysis

The method for the determination of chemical oxygen demand (COD) in water samples using LCK 314 cuvette test (Hach Lange GmbH, Berlin, Germany) is based on the oxidation of the sample with reagents delivered as ready-to-use sets, namely: potassium dichromate (VI) (CAS no. 7778-50-9), sulfuric (VI) acid (CAS no. 7664-93-9), silver (I) sulfate (CAS no. 10294-26-5) and mercury (II) sulfate (CAS no. 7783-35-9). The solution was heated at 148 ± 2 °C with a thermo-reactor LT 200 (Hach Lange GmbH, Berlin, Germany) for two hours before the determination of COD in the range of 15–150 mg/L O_2_ using a portable spectrophotometer DR 3900 (Hach Lange GmbH, Berlin, Germany) at 448 nm.

BOD (Biochemical Oxygen Demand) is a measure of the quantity of oxygen consumed by microorganisms in an aqueous sample after 5 days under conditions specified in ISO 5815-2 and DIN EN1899-2.

Measurement of total bound nitrogen (N) in water samples with cuvette tests LCK 138 is based on the oxidation of the organic and inorganic forms of nitrogen with peroxydisulphate to nitrates, which then react with 2,6-dimethilphenol (CAS no. 576-26-1) in sulfuric (VI) acid (CAS no. 7664-93-9) and phosphoric (V) acid (CAS no. 7664-38-2) media, yielding 4-nitrophenol (CAS no. 100-02-7). The solution was heated to 100 ± 2 °C (LT 200) for one hour before the determination of N in the range 1–16 mg/L at 370 nm (DR 3900).

The method for the determination of total phosphorus (P) in water samples using LCK 348 is based on the interaction of the phosphate ions with molybdate ions and antimony (CAS no. 7440-36-0) for the formation of antimonylphosphomolybdate, which was reduced by ascorbic acid (CAS no. 50-81-7) to phosphomolybdate blue and heating it for one hour at 100 ± 2 °C (LT 200) before determination of P in the range 1 mg/L to 10 mg/L at 890 nm (DR 3900). 

Total suspended solids (TSS) is a sum parameter describing the dry-weight of particles trapped by a filter. The determination method for TSS in water is based on the air-pressured filtration of the sample through a pre-weighed glass-fibre filter with specified pore size, then weighing the filter again after drying to remove all water. The gain in weight is a dry weight measure of the particulates present in the water sample (ISO 11923).

All necessary reagents (analytical grade) for BOD measurements (according to ISO 5815-2) and the determination of TSS (according to ISO 11923) are specified in the respective standard methods.

### 4.3. Multivariate Data Analysis

Principal component analysis (PCA) is a well-known statistical technique for the multivariate analysis of environmental monitoring data [42,49,50]. PCA looks for the principal components (latent factors) that describe the major variance sources present in a particular dataset [51]. To extract the latent factors explaining the major part of data variance PCA decomposes the data matrix (D) as the product of two orthogonal factor matrices U and V^T^.

(1) $$D=UV^{T}+E$$

where **D** is the data matrix of dimensions (I, J): I is the number of samples (monthly loads of all WWTPs), J is the number of variables (calculated loads for treated wastewaters). **U** is the matrix of principal component sample scores of dimension (I, N), where N is the number of principal components. **V^T^** is the matrix of loadings with dimension (N, J). **E** is the residual matrix with the same dimensions as the data matrix (**D**). By using PCA, data can be interpreted using a fewer number of principal components than the number of original variables while retaining a substantial part of the information. The two matrices **U** (scores) and **V^T^** (loadings) contain useful information about hidden relationships within the dataset and should be used for the identification of sources that contributed to the treated wastewater loads. However, the score and loadings profiles obtained by PCA could not be used as source profiles since they are orthogonal and have negative values. 

Multivariate curve resolution (MCR) decomposes the data matrix into two-factor matrices (Equation (1)) by alternating least squares optimization (ALS) [52] using as initial estimations for source composition profiles (**V^T^**) the purest sample compositions acquired in the rows of the experimental data matrix (**D**). The method provides non-negative solutions without using orthogonal constraints which leads to physically meaningful load sources. Thus, the obtained scores and loading matrixes could be used for source apportioning of treated wastewater loads. The contribution of each source (in %) to the monthly wastewater loads could be calculated using the following equation:

(2) $$p_{\mathrm{jn}}=\;\frac{\sum_{i=1}^{I}d_{\mathrm{ijn}}}{\sum_{n=1}^{N}\sum_{i=1}^{I}d_{\mathrm{ijn}}}\;\times 100$$

where $\sum_{i=1}^{I}d_{\mathrm{ijn}}$ is the sum of MCR calculated monthly loads for all WWTPs taking into consideration only the contribution of source n. The source contribution is acquired by **D^n^** matrix (**D^n^** = u_n_ v_n_^T^) obtained by the score and loading vectors of source n. The term $\sum_{n=1}^{N}\sum_{i=1}^{I}d_{\mathrm{ijn}}$ is the sum of MCR calculated monthly loads for all WWTPs taking into consideration all included in the MCR model sources (**D** = u v^T^). Before the multivariate analysis, the data matrix was either autoscaled (for PCA) or undergo min–max normalization (for MCR-ALS). More details concerning the implementation of PCA and MCR could be found in [53]. 

All multivariate analysis calculations were performed under MATLAB R2018b using PLS Toolbox 8.7 (Eigenvector Research Inc, Manson, WA, USA) and MCR-ALS Toolbox [54] which could be freely downloaded at https://mcrals.wordpress.com/.

## 5. Conclusions

The mean concentrations in the effluents of the studied WWTPs are lower than the respective limits set in the Directive 91/271/EEC for all the mandatory parameters in all the studied WWTPs throughout 2017. This shows adequate treatment regardless of occasional exceedings. The ranges of the calculated loads per p.e. for the five water quality indicators (BOD, COD, T, P and TSS) are in agreement with similar studies for wastewaters. The minimum values for N and P as well as the load ranges for BOD and especially for TSS are much lower than those for the other countries. The typical ratios mgP/mgTSS and mgBOD/mgTSS are almost twice as higher for all the Bulgarian WWTPs. The reason for such observations is the relatively low TSS loads at the inlet and the outlet of the plants. As regards to the mgBOD/mgCOD ratio, half of the Bulgarian WWTPs fall in the typical range. The higher ratio is characteristic for the Black Sea WWTPs and can be explained with the treatment of primarily domestic waters especially in the summer months and the lower range is characteristic for WWTPs in highly industrialized cities. Finally, 33% of the plants fall within the ranges of the typical ratios (mgP/mgTSS, mgBOD/mgTSS and mgBOD/mgCOD).

WWTP effluent composition, based on the load profiles of investigated Bulgarian WWTPs, shows that the average source contribution of TSS (76%) is in good agreement with the typical content of biodegradable VSS of TSS (72%), as the remaining 24% is contributed by the “refractory loads” source. The source contribution of P load is dominated by the “refractory loads” source (84%). The N load is divided by the “refractory loads” and “soluble nutrients” sources. The “suspended solids” source represents the relatively low TSS loads of WWTP effluents (as already discussed) contributes to 66% of BOD and 50% of COD load. The other half of the COD load is divided by “soluble nutrients” (28%) and “refractory loads” (22%) sources. The source load profiles of each WWTP effluent convey specific information concerning the composition and source contribution of discharged WWTP effluents. This information could be used for the management of wastewater treatment operations and the pressure and impact analysis of WWTPs on the receiving water bodies.

## Figures and Tables

**Table 1:** Basic statistics of 39 wastewater treatment plants (WWTPs) for 2017 (n = 468).

Parameter	Concentration (mg/L)					Load (kg/day)				
	Mean	Median	Min	Max	Stdev	Mean	Median	Min	Max	Stdev
**COD**	37.09	22.15	5.00	702.0	59.42	772.7	328.0	9.9	10569.0	1362.6
**BOD**	11.12	5.90	0.90	323.5	28.16	211.4	75.0	0.9	4870.5	427.6
**N**	9.68	8.69	1.40	39.50	5.89	273.1	86.3	2.7	3543.5	556.3
**P**	1.13	0.94	0.01	6.20	0.72	27.3	12.4	0.1	355.3	51.8
**TSS**	12.27	8.00	0.20	306.0	20.64	241.9	93.6	2.5	4607.0	403.5

**Table 2:** Person load in this and various studies.

Reference	Unit	COD	BOD	N	P	TSS
this study	g/p.e./day	1.3–61.7	0.2–29.5	0.36–16.3	0.03–1.45	0.21–18.8
[44]	g/person/day	–	80	13	3.2	90
[45]	g/p.e./day	36–159	17–76	4.2–18	0.68–2.5	14.2–87
[4]	g/cap/day	25–200	15–80	2–15	1–3	–

**Table 3:** Comparison between the person load in Bulgaria and other countries.

kg/Year/Person	Unit	BOD	N	P	TSS
this study	kg/p.e./yr	0.08–10.8	0.13–6.0	0.01–0.53	0.08–6.86
Brazil	kg/cap/yr	20–25	3–5	0.5–1	20–25
Egypt	kg/cap/yr	10–15	3–5	0.4–0.6	15–25
India	kg/cap/yr	10–15	–	–	–
Turkey	kg/cap/yr	10–15	3–5	0.4–0.6	15–25
US	kg/cap/yr	30–35	5–7	0.8–1.2	30–35
Denmark	kg/cap/yr	20–25	5–7	0.8–1.2	30–35
Germany	kg/cap/yr	20–25	4–6	0.7–1	30–35

**Table 4:** Calculated ratios between the chemical oxygen demand (COD), biochemical oxygen demand (BOD), P and total suspended solids (TSS) loads.

	P/TSS	BOD/TSS	BOD/COD
**Average**	0.17	1.05	0.28
**min**	0.03	0.27	0.07
**max**	0.67	4.20	0.61

## Supplementary Materials

The following are available online, Table S1: Numbers, population equivalent and daily load per p.e. of the WWTPs studied., Table S2: Number of the monthly samples from the mandatory monitoring for 2017 of the 39 studied WWTPs exceeding Directive 91/271/EEC.
