# Supplementary material for: Chemometric Assessment of Bulgarian Wastewater Treatment Plants’ Effluents
Source: Molecules. 2020 Sep 25;25(19):4408. doi: 10.3390/molecules25194408 (PMC7583790; doi:10.3390/molecules25194408)
Supplement: Supplementary file 1 [file molecules-25-04408-s001.pdf]

Electronic Supplementary Material
Article

Chemometric assessment of Bulgarian wastewater treatment plants' effluents

Galina Yotova [1], Tony Venelinov [2] and Stefan Tsakovski [1,*]

[1] Faculty of Chemistry and Pharmacy, Sofia University "St. Kliment Ohridski", 1164 Sofia, Bulgaria; G.Yotova@chem.uni-sofia.bg
[2] Faculty of Hydraulic Engineering, University of Architecture, Civil Engineering and Geodesy, 1046 Sofia, Bulgaria; TVenelinov_fhe@uacg.bg
* Correspondence: STsakovski@chem.uni-sofia.bg; Tel.: +359-2-8161426

Table S1. Numbers, population equivalent and daily load per p.e. of the WWTPs studied.

Number	Sampling location	Population equivalent (p.e.)	Treatment facilities[1]	Load (g/p.e./day)				
				COD	BOD	N	P	TSS
1	Montana	98618	4	3.70	2.26	2.04	0.19	2.38
2	Pleven	657000	2	1.74	0.42	0.76	0.07	0.72
3	Lovech	85700	4	2.63	0.44	1.00	0.27	0.40
4	Troyan	80000	4	2.23	0.39	0.67	0.03	0.54
5	Sevlievo	54000	4	2.41	0.90	0.75	0.14	0.21
6	Gabrovo	99780	4	3.54	0.94	0.95	0.24	2.90
7	Veliko Tarnovo	165625	2	3.70	0.25	0.58	0.15	0.31
8	Gorna Oryahovitsa	102000	4	2.10	0.41	0.66	0.10	1.53
9	Popovo	37720	3	6.71	1.60	0.78	0.18	1.82
10	Razgrad	78200	2	9.63	2.02	1.30	0.17	2.96
11	Shumen	132000	4	14.28	6.65	1.93	0.19	4.91
12	Shabla	360	2	150.33	60.56	100.26	10.40	35.97
13	Kavarna	3583	2	39.19	11.88	16.31	1.45	3.98
14	Balchik	22200	4	2.00	0.75	0.97	0.09	0.59
15	Albena	19000	2	8.37	2.85	2.84	0.29	1.52
16	Zlatni Pyasatsi	72122	2	6.87	1.43	0.90	0.12	2.09
17	Varna	450000	4	7.89	1.57	1.53	0.15	3.25
18	Beloslav	14968	4	1.54	0.23	0.49	0.10	0.80
19	Devnya	23300	2	8.82	2.09	6.23	0.55	5.62
20	Provadiya	16770	4	4.75	0.94	1.47	0.19	2.62
21	Byala-Obzor	40000	4	3.88	0.76	1.47	0.10	1.10
22	Nesebar-Ravda-Slanchev bryag	222000	4	4.80	1.02	0.96	0.07	1.30
23	Pomorie	56626	4	7.87	0.99	1.76	0.17	2.32
24	Burgas	397700	4	3.28	0.45	0.78	0.04	0.99
25	Meden Rudnik	49000	4	3.04	0.38	0.51	0.09	1.19
26	Kiten	28748	2	6.93	1.35	1.93	0.16	1.53
27	Lozenets	3000	4	61.71	29.46	4.38	0.65	18.78

28	Nova Zagora	59673	2	8.26	3.75	2.27	0.24	2.95
29	Stara Zagora	256300	4	5.32	2.48	2.07	0.12	2.25
30	Kazanlak	80000	4	7.55	1.63	2.13	0.31	2.94
31	Sopot	25000	4	1.33	0.40	0.36	0.08	0.30
32	Haskovo	95637	4	9.48	2.51	3.65	0.35	8.19
33	Plovdiv	596000	2	7.96	1.92	3.26	0.44	1.77
34	Pazardzhik	150000	2	3.58	1.15	4.29	0.45	1.01
35	Blagoevgrad	87520	4	5.37	1.79	1.75	0.37	2.35
36	Dupnitsa	55000	2	5.75	1.76	1.13	0.28	1.35
37	Samokov	40000	4	8.08	2.16	2.13	0.25	2.32
38	Pernik	160000	2	2.45	1.24	1.30	0.17	0.58
39	Sofia	1833333	4	3.04	0.88	1.61	0.10	0.47

[1]Treatment facilities are marked as follows: 1 – mechanical treatment; 2 – mechanical and biological treatment; 3 – mechanical, biological treatment and nitrogen removal facility; 4 – mechanical, biological treatment, nitrogen removal facility and chemical precipitation of phosphorus.

Table S2. Number of the monthly samples from the mandatory monitoring for 2017 of the 39 studied WWTPs exceeding Directive 91/271/EEC.

**WWTP[1]**	**Sampling location**	**Samples exceeding Directive 91/271/EEC[2]**				
		**COD**	**BOD**	**N**	**P**	**TSS**
11	Shumen	3	3	8	7	3
12	Shabla	-	-	7	3	-
13	Kavarna	-	-	12	11	-
19	Devnya	-	-	1	-	-
26	Kiten	-	-	2	-	-
27	Lozenets	6	9	5	4	7
28	Nova Zagora	-	3	-	-	-
32	Haskovo	-	-	1	1	1
33	Plovdiv	-	-	11	11	-
34	Pazardzhik	-	-	12	12	-
36	Dupnitsa	-	-	-	1	-
38	Pernik	-	-	1	6	-
Total		9	15	60	56	11

[1]For all the remaining WWTPs no exceedings are observed; [2]The concentration limits (mg/L) according to Directive 91/271/EEC are as follows: COD (125 mg/L); BOD (25 mg/L); N (10 mg/L for more than 100000 p.e., 15 mg/L for 10000-100000 p.e.); P (1 mg/L for more than 100000 p.e., 2 mg/L for 10000-100000 p.e.); TSS (35 mg/L for more than 10000 p.e., 60 mg/L for 2000-10000 p.e.).
